# Long-term virological suppression on first-line efavirenz + tenofovir + emtricitabine/lamivudine for HIV-1

**DOI:** 10.1097/QAD.0000000000002126

**Published:** 2019-01-03

**Keywords:** antiretroviral therapy, viral failure, viral rebound, viral suppression, virological control

## Abstract

**Objectives:**

Evaluate long-term rates of virological failure and treatment interruption for people living with HIV (PLWHIV) with viral suppression on first-line efavirenz + tenofovir disoproxil fumarate + emtricitabine/lamivudine (EFV + TDF + FTC/3TC), and compare these according to patient characteristics.

**Methods:**

PLWHIV enrolled in the Collaboration of Observational HIV Epidemiological Research Europe cohort collaboration, who started first-line EFV + TDF + FTC/3TC at age at least 16 years and had viral suppression (<200 copies/ml) within 9 months were included. Rates of virological failure (≥200 copies/ml) and (complete) treatment interruption were estimated according to years since initial suppression. We used Poisson regression to examine associations of baseline characteristics with rates of virological failure or treatment interruption.

**Results:**

Among 19 527 eligible PLWHIV with median (interquartile range) follow-up 3.7 (2.0–5.6) years after initial viral suppression, the estimated rate of the combined incidence of virological failure or treatment interruption fell from 9.0/100 person-years in the first year to less than 4/100 person-years beyond 3 years from suppression; considering only those remaining on EFV + TDF + FTC/3TC, the combined rate dropped from 8.2/100 person-years in the first year to less than 3.5/100 person-years beyond 3 years. PLWHIV with injecting drug-related or heterosexual transmission were at higher risk of virological failure or treatment interruption, as were those of Black ethnicity. PLWHIV aged less than 35 years were at higher risk of virological failure and treatment interruption.

**Conclusion:**

PLWHIV starting first-line EFV + TDF + FTC/3TC had low rates of virological failure and treatment interruption up to 10 years from initial suppression. Demographic characteristics can be used to identify subpopulations with higher risks of these outcomes.

## Introduction

The combination of efavirenz (EFV), tenofovir disoproxil fumarate (TDF) and emtricitabine (FTC) or lamivudine (3TC) was established as preferred first-line antiretroviral therapy (ART) for HIV in 2013 WHO guidelines [1]. However, first-line EFV is no longer the preferred choice in most patients [2] because of the availability of new combinations with greater efficacy and fewer side effects and emergence of high levels of transmitted resistance to nonnucleoside/nucleotide reverse transcriptase inhibitors in some low/middle income countries (LMICs) [3]. Despite reductions in newly diagnosed people living with HIV (PLWHIV) starting EFV + TDF + FTC/3TC, large numbers remain on this regimen [4] and so there is a need to evaluate its long-term effectiveness.

An analysis from the UK Collaborative HIV Cohort (UK CHIC) Study showed that PLWHIV on ART regimens with initial viral suppression have annual rates of virological rebound (200 copies/ml threshold) that decrease from around 9/100 person-years in the first year to less than 3/100 person-years after some years on treatment [5]; the authors projected that some PLWHIV would maintain suppression for decades without treatment change. However, few studies have evaluated long-term viral suppression using a single combination.

We estimated rates of virological failure, treatment interruption and treatment switches for PLWHIV with initial viral suppression on first-line EFV + TDF + FTC/3TC within a multinational collaboration of European HIV cohort studies. We evaluated demographic and clinical risk factors for these events.

## Methods

We analysed data, merged in June 2015, from 20 cohorts in the Collaboration of Observational HIV Epidemiological Research Europe (COHERE) [6]. Additional data were added from the UK CHIC September 2016 dataset, to align last recorded follow-up with other cohorts.

PLWHIV were included if they were ART-naïve at cohort enrolment and started first-line EFV + TDF + FTC/3TC at age at least 16 years with viral suppression (defined as one measurement undetectable or <200 copies/ml) within 9 months. Initial regimen was ignored if it changed within 1 week of first treatment. PLWHIV were excluded if their last viral load measurement was less than 9 months after starting ART, if treatment was interrupted before viral suppression or if at least one pre-ART viral load measurement was either undetectable or ≤50 copies/ml within 1 year prior to starting ART (to remove those who may have started ART before the date recorded). COHERE cohorts with fewer than 20 PLWHIV meeting the inclusion criteria were dropped.

### Statistical analysis

Follow-up started at the date of viral suppression. Virological failure was defined as one measurement ≥ 200 copies/ml to allow consistency of analysis across cohorts and over the timespan considered. Treatment interruption was defined as cessation of all ART, but interruptions of up to 1 week were ignored. We also estimated rates of virological failure whilst on EFV + TDF + FTC/3TC, of complete interruption of ART directly from treatment with EFV + TDF + FTC/3TC and of switching from EFV + TDF + FTC/3TC to any other regimen.

Piecewise exponential time-to-event models were used to estimate event rates, which were estimated for yearly intervals from initial viral suppression, with a single rate estimated for follow-up more than 9 years. Rates were first estimated without adjustment for patient characteristics. For virological failure and treatment interruption, cause-specific piecewise exponential models were fitted with censoring for the other event (i.e. only the first virological failure *or* interruption event was counted): these were used to estimate the rates and cumulative incidence of each event, accounting for the competing risk of the other [7].

We estimated adjusted associations of the virological failure and treatment interruption outcomes with patient sex, mode of acquisition (MSM [reference], female heterosexual, male heterosexual, female IDU, male IDU), ethnicity (white [reference], Black, Asian, other), prior AIDS diagnosis, baseline CD4^+^ cell count (0–200 [reference], 200–350, 350–500, >500 cells/μl) and viral load (0–20k, 20k–100k [reference], 100k–500k, >500k copies/ml), time-updated age (<25, 25–35, 35–45 [reference], 45–55, >55 years), year of starting ART (2002–2004, 2005–2006, 2007–2008, 2009–2010 [reference], 2011–2012, 2013–2014) and cohort. For five cohorts, ethnicity was not recorded so this variable was set to reference (i.e. ‘white’) for the purpose of multivariable analysis. For categorical variables, the group with highest frequency was chosen as reference. Baseline CD4^+^ cell counts and viral load were defined as the last measurement obtained within the 6-month period before ART start. Follow-up was censored at 6 months after last recorded viral load or at death. The adjusted analyses were conducted without censoring at switch to other ART regimen.

For analyses adjusted for patient characteristics, full covariate data were available in 83.0% of cases (ignoring ethnicity for cohorts without this information recorded). Multiple imputation using chained equations was implemented using the Stata ‘ice’ package [8], with 17 imputed datasets for each event [9]. Imputation models included log event times and indicators for cohorts and events. Predictive mean matching was employed for baseline CD4^+^, viral load and age. Imputation models used square root CD4^+^ cell counts and log_10_ viral load. MSM status, sex and IDU status were imputed separately; these factors were then combined for analysis models with IDU status considered the primary mode of acquisition in PLWHIV who were also MSM. PLWHIV with transfusion-related or ‘other’ acquisition were excluded due to low numbers.

## Results

The study population included 19 527 PLWHIV (Fig. S1). The majority mode of acquisition was MSM (59.6%). Where known, white ethnicity was most common (70.2%) with 20.6% Black and 4.2% Asian ethnicities (further details in Tables S1 and S2).

**Fig. 1:**
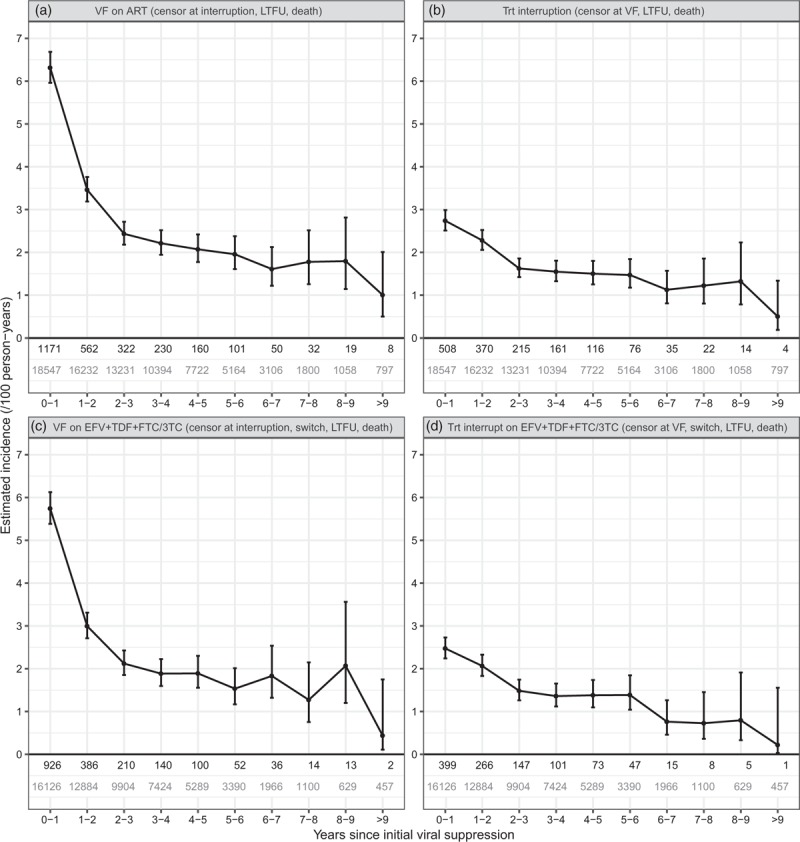
Plots of unadjusted incidence rate estimates (per 100 person-years) according to years since initial viral suppression for virological failure (VF) and treatment interruption (censoring at VF) whilst on any antiretroviral therapy [(a) and (b)], and whilst on efavirenz + tenofovir + emtricitabine/lamivudine [(c) and (d), respectively].

Unadjusted incidence rates of virological failure and ART interruption according to years since initial viral suppression are shown in Fig. 1a and b. For these analyses (counting only the first event), there were 2655 (13.6%) virological failure events and 1521 (7.8%) treatment interruption events, and median (interquartile range) follow-up was 3.7 (2.0–5.6) years. For analyses restricted to those remaining on EFV + TDF + FTC/3TC, incidence rates are shown in Fig. 1c and d: there were 1879 (9.6%) virological failure outcomes and 1062 (5.4%) treatment interruption outcomes. Results in supplementary material show rates of switching from EFV + TDF + FTC/3TC to any other ART regimen, the combined incidence of ‘virological failure or treatment interruption’ (Fig. S2), and cumulative incidence functions for virological failure and ART interruption (Fig. S3).

Virological failure defined as any viral load observation ≥ 200 copies/ml. Loss to follow-up (LTFU) defined in terms of virological monitoring, with censoring 6 months after last recorded viral load measurement. Error bars show 95% confidence interval for estimates. The number of events for each time period is given directly below each plot, whilst the ‘person-years at risk’ for each period is given below that (gray text).

Fifty-five PLWHIV with transfusion-acquired HIV and 262 with ‘other’ acquisition were excluded from multivariable analyses. Adjusted associations of patient characteristics with virological failure and treatment interruption are shown in Table 1. MSM had lowest rates of virological failure and treatment interruption while IDU had markedly higher rates of treatment interruption. Black ethnicity was associated with higher rates of virological failure and treatment interruption. A prior AIDS diagnosis was associated with higher rates of virological failure but lower rates of treatment interruption. Baseline CD4^+^ cell count more than 200 cells/μl was associated with lower rate of virological failure, but those with baseline CD4^+^ above 500 cells/μl had higher rates of treatment interruption. Rates of virological failure increased with increasing baseline viral load, but there was little evidence that baseline viral load was associated with treatment interruption. Rates of both virological failure and treatment interruption declined in later compared with earlier calendar years of starting ART. Age below 35 years was associated with a higher rate of virological failure and of treatment interruption.

## Discussion

Amongst PLWHIV enrolled in a large collaboration of European cohort studies starting first-line EFV + TDF + FTC/3TC, rates of virological failure and treatment interruption declined over 3 years following initial virological suppression before stabilizing at low levels: the subsequent combined incidence rate of virological failure or treatment interruption was below 4/100 person-years for PLWHIV remaining on ART, and was below 3.5/100 person-years considering only those on the EFV + TDF + FTC/3TC regimen.

The regimen included in this analysis, EFV + TDF + FTC/3TC, is no longer preferred first-line ART in most patients: WHO now recommends dolutegravir-based ART [2] following evidence that this has improved efficacy and reduced side effects [10]. Tenofovir alafenamide may also have a better side effect profile than TDF in some combinations [11]. However, whilst agreements are in place to provide dolutegravir-based ART at low cost in LMICs [4], EFV + TDF + FTC/3TC is available as a low-cost generic option worldwide and there is not strong evidence for an individual-level benefit of switching off this regimen in virologically suppressed patients [4]. At present, there are also concerns regarding the use of dolutegravir in women who may become pregnant [12].

We found lower rates of both virological failure and treatment interruption for PLWHIV on first-line EFV + TDF + FTC/3TC in comparison to UK patients starting any 3 + drug ART [5], for whom a combined incidence of around 12.5/100 person-years was reported in the first year from baseline (*c.* 9.0/100 person-years) dropping to less than 6/100 person-years beyond 3 years (*c.* <4/100 person-years). Long-term studies have not yet been published regarding the durability of viral suppression on first-line dolutegravir-based ART, but as there is evidence from trials of superior viral suppression on dolutegravir vs. EFV at 48, 96 and 144 weeks [13] it is likely that our results reflect an upper limit on the virological failure rates that would be expected for equivalent patients on dolutegravir-based ART.

Black ethnicity was associated with virological failure and treatment interruption, which is consistent with the findings of O’Connor *et al.*[5] for the United Kingdom. Non-MSM groups were also at higher risk of these events, with particularly strong associations for IDUs as found previously [14]. Rates of virological failure among non-white and non-MSM individuals may vary between countries and healthcare settings, but these findings reinforce the need to identify subpopulations with worse outcomes on ART [15] and understand the underlying causes. Both ethnicity and mode of acquisition are associated with social and economic factors which themselves may vary between cohorts. Age less than 35 was associated with higher rates of virological failure and treatment interruption, consistent with previous findings in both high income [5,14] and LMIC [16] settings. Differences in rates of virological failure between demographic groups are likely to be driven by adherence [17].

Consistent with previous literature [5,18] viral load before starting treatment was associated with rates of virological failure on treatment, whilst baseline CD4^+^ cell count more than 200 cells/μl was associated with a lower rate of virological failure on treatment [19]. PLWHIV with the highest baseline CD4^+^ cell counts (>500 cells/μl) had highest rate of treatment interruption, consistent with previous studies [19,20], which could reflect differences in behaviour and clinical counselling for PLWHIV at lower immediate risk of HIV-related morbidity. Most data in this analysis were from the period before European guidelines recommended starting ART in all PLWHIV irrespective of CD4^+^ cell count.

Rates of virological failure and treatment interruption for PLWHIV declined in later compared with earlier calendar years of ART initiation. This may be linked to a reduction in pill count as combination tablets became available [21], but a limitation of our analysis is that we do not have detailed information on combination dosing (e.g. number of pills/day). Another limitation is that we cannot determine whether treatment switching from first-line regimen was driven by side effects.

We have quantified long-term virological suppression achieved using first-line EFV + TDF + FTC/3TC across a large multinational cohort collaboration. This regimen remains in use worldwide, so the low failure rate with sustained virological suppression for up to a decade on treatment is encouraging. The substantial differences in rates of virological failure and treatment interruption according to demographic and clinical characteristics may be useful for targeted monitoring and adherence interventions.

## Acknowledgements

Contribution of working group members: A.P., D.D. and J.S. developed the research question and study design. D.B. coordinated collaboration between cohorts for the project. O.S. performed the statistical analyses and drafted the initial text of the article. All other working group members contributed data to the analysis, and all working group members were involved in the interpretation of results and final text of the article.

**The Long-Term Virological Suppression Working Group:** Oliver T. Stirrup^1^, Jonathan Sterne^2^, David T. Dunn^1^, Katharina Grabmeier-Pfistershammer^3^, Vasileios Papastamopoulos^4^, Marie-Anne Vandenhende^5,6^, Ferdinand Wit^7^, Kholoud Porter^1^, Barbara Gunsenheimer-Bartmeyer^8^, Inma Jarrin^9^, Federico Garcia^10^, Gerd Fätkenheuer^11^, Niels Obel^12^, Anna Schultze^13^, Andrea Antinori^14^, Francesca Ceccherini-Silberstein^15^, Cristina Mussini^16^, Geneviève Chêne^5,17^, Bastian Neesgaard^18^, Antonella Castagna^19^, Roger Kouyos^20^, Stéphane De Wit^21^, Anders Sönnerborg^22^, Caroline Sabin^1^, Dolores Merino^23^, Diana Barger^5,17^, Andrew Phillips^1^.

^1^Institute for Global Health, University College London, London; ^2^Population Health Sciences, Bristol Medical School, Bristol, UK; ^3^Medical University of Vienna, Vienna, Austria; ^4^Evaggelismos General Hospital, Athens, Greece; ^5^University Bordeaux, Inserm, Bordeaux Population Health Research Center, U1219; ^6^Bordeaux University Hospital, Hôpital Saint-André, Bordeaux, France; ^7^Academic Medical Center, Amsterdam, The Netherlands; ^8^Robert Koch Institute, Berlin, Germany; ^9^Instituto de Salud Carlos III, Madrid; ^10^Clinical Microbiology & Infectious Diseases Unit, Hospital Universitario San Cecilio, Instituto de Investigación Ibs. Granada, Granada, Spain; ^11^University Hospital Cologne, Cologne, Germany; ^12^Copenhagen University Hospital, Rigshospitalet, Copenhagen, Denmark; ^13^University College London, London, UK; ^14^INMI, Lazzaro Spallanzani; ^15^Department of Experimental Medicine and Surgery, University of Rome Tor Vergata, Rome; ^16^Clinic of Infectious Diseases, University Hospital, University of Modena and Reggio Emilia, Modena, Italy; ^17^ISPED, CHU Bordeaux, Bordeaux, France; ^18^CHIP, University of Copenhagen, Copenhagen, Denmark; ^19^Clinic of Infectious Diseases, Vita-Salute San Raffaele University, Milan, Italy; ^20^Division of Infectious Diseases and Hospital Epidemiology, University Hospital Zurich, University of Zurich, Zürich, Switzerland; ^21^Department of Infectious Diseases, St Pierre University Hospital, Brussels, Belgium; ^22^Karolinska Institutet, Karolinska University Hospital, Stockholm, Sweden; ^23^Unidad de Gestión Clínica de Enfermedades Infecciosas, Complejo Hospitalario de Huelva, Huelva, Spain.

**Steering Committee – Contributing Cohorts:** Ali Judd (AALPHI), Robert Zangerle (AHIVCOS), Giota Touloumi (AMACS), Josiane Warszawski (ANRS CO1 EPF/ANRS CO11 OBSERVATOIRE EPF), Laurence Meyer (ANRS CO2 SEROCO), François Dabis (ANRS CO3 AQUITAINE), Murielle Mary Krause (ANRS CO4 FHDH), Jade Ghosn (ANRS CO6 PRIMO), Catherine Leport (ANRS CO8 COPILOTE), Linda Wittkop (ANRS CO13 HEPAVIH), Peter Reiss (ATHENA), F.W. (ATHENA), Maria Prins (CASCADE), Heiner Bucher (CASCADE), Diana Gibb (CHIPS), G.F. (Cologne-Bonn), Julia Del Amo (CoRIS), N.O. (Danish HIV Cohort), Claire Thorne (ECS, NSHPC), Amanda Mocroft (EuroSIDA), Ole Kirk (EuroSIDA), Christoph Stephan (Frankfurt), Santiago Pérez-Hoyos (GEMES-Haemo), Osamah Hamouda (German ClinSurv), B.G.-B. (German ClinSurv), Nikoloz Chkhartishvili (Georgian National HIV/AIDS), Antoni Noguera-Julian (CORISPE-cat), A.A. (ICC), Antonella d’Arminio Monforte (ICONA), Norbert Brockmeyer (KOMPNET), Luis Prieto (Madrid PMTCT Cohort), Pablo Rojo Conejo (CORISPES-Madrid), Antoni Soriano-Arandes (NENEXP), Manuel Battegay (SHCS), R.K. (SHCS), C.M. (Modena Cohort), Jordi Casabona (PISCIS), Jose M. Miró (PISCIS), A.C. (San Raffaele), Deborah Konopnick (St. Pierre Cohort), Tessa Goetghebuer (St Pierre Paediatric Cohort), A.S. (Swedish InfCare), Carlo Torti (The Italian Master Cohort), C.S. (UK CHIC), Ramon Teira (VACH), Myriam Garrido (VACH), David Haerry (European AIDS Treatment Group).

**Executive Committee:** S.D.W. (Chair, St. Pierre University Hospital), Jose Mª Miró (PISCIS), Dominique Costagliola (FHDH), Antonella d’Arminio-Monforte (ICONA), A.C. (San Raffaele), Julia del Amo (CoRIS), Amanda Mocroft (EuroSida), Dorthe Raben (Head, Copenhagen Regional Coordinating Centre), G.C. (Head, Bordeaux Regional Coordinating Centre). Paediatric Cohort Representatives: Ali Judd, Pablo Rojo Conejo.

**Regional Coordinating Centres:** Bordeaux RCC: D.B., Christine Schwimmer, Monique Termote, Linda Wittkop; Copenhagen RCC: Casper M. Frederiksen, Dorthe Raben, Rikke Salbøl Brandt.

**Project Leads and Statisticians:** Juan Berenguer, Julia Bohlius, Vincent Bouteloup, Heiner Bucher, Alessandro Cozzi-Lepri, François Dabis, Antonella d’Arminio Monforte, Mary-Anne Davies, Julia del Amo, Maria Dorrucci, D.T.D., Matthias Egger, Hansjakob Furrer, Marguerite Guiguet, Sophie Grabar, Ali Judd, Ole Kirk, Olivier Lambotte, Valériane Leroy, Sara Lodi, Sophie Matheron, Laurence Meyer, Jose Mª Miró, Amanda Mocroft, Susana Monge, Fumiyo Nakagawa, Roger Paredes, A.P., Massimo Puoti, Eliane Rohner, Michael Schomaker, Colette Smit, J.S., Rodolphe Thiebaut, Claire Thorne, Carlo Torti, Marc van der Valk, Linda Wittkop.

The COHERE study group has received unrestricted funding from Agence Nationale de Recherches sur le SIDA et les Hépatites Virales (ANRS), France; HIV Monitoring Foundation, The Netherlands; and the Augustinus Foundation, Denmark. The research leading to these results has received funding from the European Union Seventh Framework Programme (FP7/2007–2013) under EuroCoord grant agreement no 260694. The group has also received project-specific funding from UK Medical Research Council (Award Number 164587). A list of the funders of the participating cohorts can be found at www.COHERE.org.

### Conflicts of interest

There are no conflicts of interest.

## Supplementary Material

Supplemental Digital Content

## Figures and Tables

**Table 1:** Adjusted (for other characteristics in the table) associations (incidence rate ratios) of patient characteristics with virological failure and treatment interruption.

			VF on ART (censor at interruption, LTFU, death)			Treatment interruption (censor at VF, LTFU, death)		
		*n* Patients	*n* Events	IRR^a^ (95% CI)	*P* value^*^	*n* Events	IRR^a^ (95% CI)	*P* value^*^
ART regimen	On FTC	17 945	2256	1 [Reference]	0.140	1322	1 [Reference]	0.710
	On 3TC	1265	340	1.13 (0.96, 1.34)		165	1.05 (0.83, 1.32)	
Mode of acquisition^b^	MSM	12 399	1467	1 [Reference]	<0.001	825	1 [Reference]	<0.001
	Hetero (female)	2750	453	1.19 (1.05, 1.35)		271	1.17 (0.99, 1.39)	
	Hetero (male)	3486	577	1.32 (1.18, 1.47)		303	1.26 (1.08, 1.47)	
	IDU (female)	123	17	1.15 (0.70, 1.89)		20	3.18 (2.00, 5.04)	
	IDU (male)	451	82	1.53 (1.21, 1.94)		68	3.13 (2.40, 4.08)	
Ethnicity^b^^,^^c^	White	8226	1015	1 [Reference]	<0.001	627	1 [Reference]	<0.001
	Asian	484	63	1.12 (0.86, 1.45)		32	0.85 (0.59, 1.23)	
	Black	2363	435	1.33 (1.16, 1.53)		331	1.54 (1.29, 1.82)	
	Other	575	56	0.83 (0.63, 1.09)		55	1.03 (0.78, 1.37)	
Prior AIDS Dx	No	16 803	2124	1 [Reference]	0.001	1298	1 [Reference]	0.059
	Yes	2407	472	1.20 (1.08, 1.34)		189	0.85 (0.72, 1.01)	
Baseline CD4^+^ cell count (cells/μl)^b^	0–200	8879	1417	1 [Reference]	0.005	673	1 [Reference]	<0.001
	200–350	6432	838	0.90 (0.82, 0.99)		514	0.97 (0.85, 1.10)	
	350–500	2707	238	0.77 (0.67, 0.90)		199	1.17 (0.98, 1.39)	
	Over 500	1191	103	0.89 (0.72, 1.11)		101	1.60 (1.28, 2.01)	
Baseline VL (copies/ml)^b^	0–20k	4580	416	0.72 (0.64, 0.82)	<0.001	362	0.88 (0.76, 1.01)	0.262
	20k–100k	6696	824	1 [Reference]		534	1 [Reference]	
	100k–500k	6130	997	1.28 (1.16, 1.42)		456	0.98 (0.86, 1.12)	
	Over 500k	1805	358	1.53 (1.34, 1.74)		135	1.03 (0.85, 1.26)	
Start of ART	2002–2004	847	259	1.64 (1.35, 2.01)	<0.001	137	1.52 (1.16, 2.00)	0.009
	2005–2006	2000	460	1.34 (1.18, 1.53)		226	1.15 (0.96, 1.37)	
	2007–2008	4137	683	1.14 (1.03, 1.27)		387	1.10 (0.96, 1.27)	
	2009–2010	6058	732	1 [Reference]		443	1 [Reference]	
	2011–2012	4763	381	0.85 (0.75, 0.97)		247	0.94 (0.80, 1.11)	
	2013–2014	1405	81	0.86 (0.68, 1.09)		47	0.78 (0.57, 1.07)	
Time-updated age (years)^d^	Under 25	912	118	1.10 (0.87, 1.38)	0.007	102	1.90 (1.48, 2.44)	<0.001
	25–35	5613	765	1.16 (1.05, 1.28)		478	1.19 (1.04, 1.35)	
	35–45	7127	982	1 [Reference]		534	1 [Reference]	
	45–55	3933	513	0.97 (0.88, 1.08)		279	0.89 (0.78, 1.02)	
	Over 55	1625	218	0.95 (0.82, 1.09)		94	0.79 (0.64, 0.96)	

3TC, lamivudine; ART, antiretroviral therapy; CI, confidence interval; Dx, diagnosis; FTC, emtricitabine; IRR, incidence rate ratio; LTFU, lost to follow-up; VF, virological failure; VL, viral load.

^a^Estimated using Poisson regression models with adjustment for cohort and all variables listed in this table.

^b^Multiple imputation used for missing data.

^c^Multiple imputation not used for ethnicity in cohorts with no data on this variable: ethnicity was set to reference and these cohorts have not been included in ethnicity rows of *n* patients and *n* events in this table.

^d^*n* Patients and *n* events are given according to baseline age at start of ART.

^*^Univariate or multivariate Wald test.
