# Supplementary material for: Long-term virological suppression on first-line efavirenz + tenofovir + emtricitabine/lamivudine for HIV-1
Source: AIDS. 2019 Jan 3;33(4):745–51. doi: 10.1097/QAD.0000000000002126 (PMC6410964; doi:10.1097/QAD.0000000000002126)
Supplement: Supplemental Digital Content [file aids-33-745-s001.pdf]

Figure S1 Flow chart of the study population.

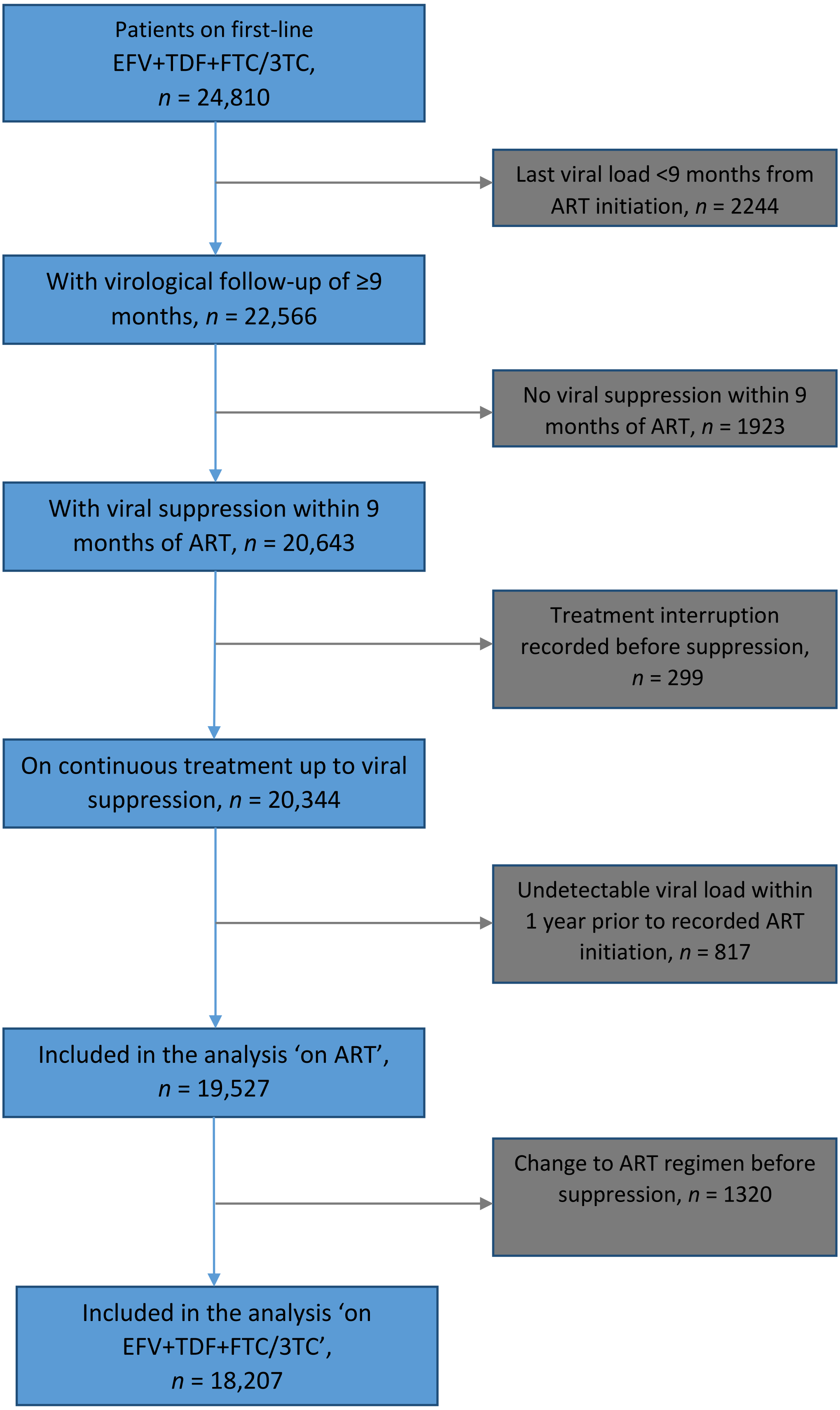

Figure S2 Plots of unadjusted incidence rate estimates (/100 person-years) according to years since initial viral suppression for (a) treatment (Trt) switch off efavirenz + tenofovir + emtricitabine/lamivudine to any other antiretroviral therapy regimen, and (b) composite endpoint of VF or complete Trt interruption. VF defined as any viral load (VL) observation ≥200 copies/mL. Loss to follow-up (LTFU) defined in terms of virological monitoring, with censoring 6 months after last recorded VL measurement. Error bars show 95%CI for estimates. Below each plot, the number of events for each time period is given in red, whilst the ‘person-years at risk’ for each period is given in green.

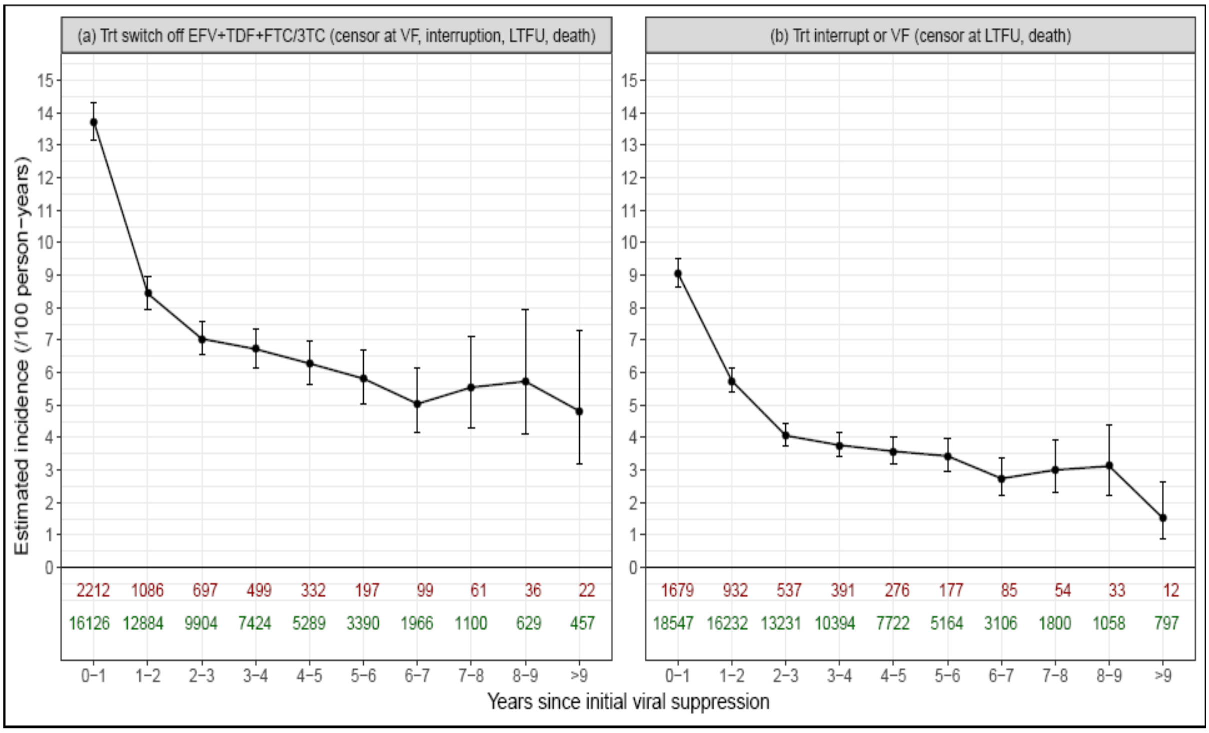

Figure S3 Stacked cumulative incidence functions for virological failure (green fill) and antiretroviral therapy (ART) interruption (red fill) estimated using a competing risks approach. Upper limit of red area represents cumulative incidence function for composite endpoint of virological failure or ART interruption.

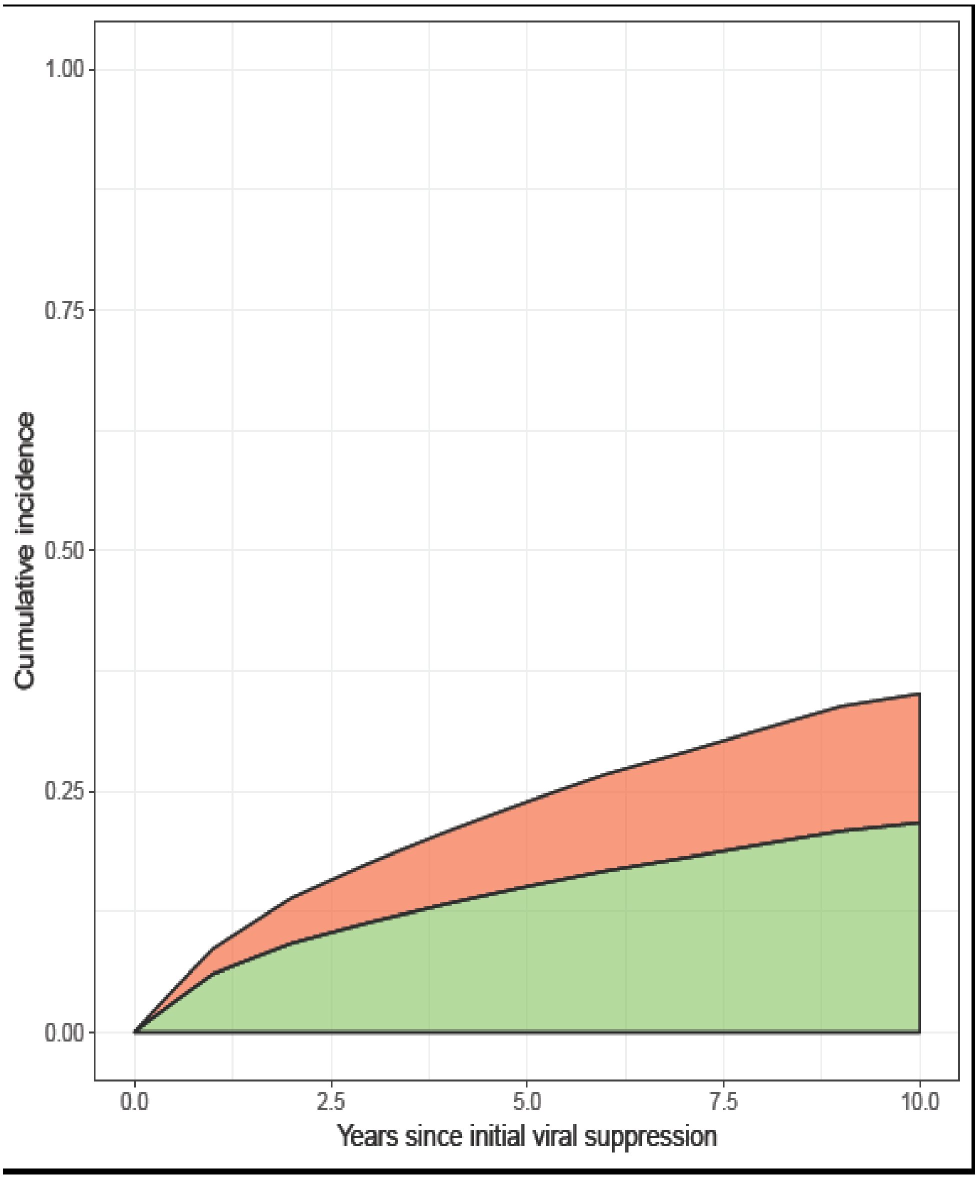

Table S1 Summary of demographic and antiretroviral therapy (ART) information in study population of people living with HIV (PLWHIV) starting first-line ART on efavirenz + tenofovir + emtricitabine/lamivudine regimen with viral suppression within 9 months (*n*=19,527).

	N (%) or median (IQR)
Mode of acquisition	
MSM	11,646 (59.6)
Hetero. (female)	2554 (13.1)
Hetero. (male)	3250 (16.6)
IDU (female)	113 (0.6)
IDU (male)	413 (2.1)
Transfusion	55 (0.3)
Other/unknown	1496 (7.7)
Ethnicity	
White	7927 (40.6)
Asian	477 (2.4)
Black	2331 (11.9)
Other	565 (2.9)
Missing/unknown	8227 (42.1)
ART regimen	
On FTC	18,231 (93.4)
On 3TC	1296 (6.6)
Year of starting ART	
2002–2004	866 (4.4)
2005–2006	2058 (10.5)
2007–2008	4219 (21.6)
2009–2010	6148 (31.5)
2011–2012	4822 (24.7)
2013–2014	1414 (7.2)
Prior AIDS Dx	2469 (12.6)
Age at baseline (years)	39 (32–46)
CD4 at baseline (cells/µL)*	218 (28–324)
VL at baseline (copies/mL) †	70,300 (21,400– 183,000)
Follow-up for analysis (years)	3.7 (2.0–5.6)

*Available in 18,604 PLWHIV. †Available in 18,182 PLWHIV. 3TC, lamivudine; FTC, emtricitabine; IDU, injecting drug user; MSM, men who have sex with men; VL, viral load.

Table S2 Numbers of people living with HIV (PLWHIV) in the total study population (*n*=19,527) according to cohort.

Cohort	Country	*n* (%)
Austrian HIV Cohort Study (AHIVCOS)	Austria	380 (1.9)
Athens Multicenter AIDS Cohort Study (AMACS)	Greece	603 (3.1)
Aquitaine Cohort*	France	207 (1.1)
AIDS Therapy Evaluation in the Netherlands (ATHENA) Cohort*	Netherlands	4138 (21.2)
Bonn cohort	Germany	132 (0.7)
Concerted Action on SeroConversion to AIDS and Death in Europe (CASCADE)*	Multinational	203 (1)
ClinSurv HIV Cohort Study (Germany)*	Germany	1660 (8.5)
Cohort of the Spanish HIV Research Network (CoRIS)*	Spain	1568 (8)
Cologne cohort	Germany	224 (1.1)
Danish HIV Cohort	Denmark	525 (2.7)
EuroSIDA	Multinational	319 (1.6)
San Raffaele Hospital cohort (Italy)	Italy	198 (1)
ICC (Italy)	Italy	299 (1.5)
Italian Cohort of Antiretroviral Naïve Patients (ICONA)	Italy	693 (3.5)
Modena cohort	Italy	165 (0.8)
Swiss HIV Cohort Study (SHCS)	Switzerland	866 (4.4)
St. Pierre , Brussels Cohort	Belgium	110 (0.6)
Swedish InfCare HIV Cohort Study	Sweden	866 (4.4)
UK Collaborative HIV Cohort (UK CHIC) Study	UK	6081 (31.1)
Spanish VIH/ACH (VACH) Cohort	Spain	290 (1.5)

*Cohort missing data on ethnicity in all patients.
